# Past Gaming Experience and Cognition as Selective Predictors of Novel Game Learning Across Different Gaming Genres

**DOI:** 10.3389/fpsyg.2020.00786

**Published:** 2020-05-26

**Authors:** Evan T. Smith, Bhargavi Bhaskar, Alex Hinerman, Chandramallika Basak

**Affiliations:** Center for Vital Longevity, The University of Texas at Dallas, Dallas, TX, United States

**Keywords:** video games, genres, learning, life span, working memory, gaming habits

## Abstract

Past experience with video games and cognitive abilities have been hypothesized to independently facilitate a greater ability to learn new video games and other complex tasks. The present study was conducted to examine this “learning to learn” hypothesis. We examined the predictive effects of gaming habits (e.g., self-identification as a “gamer,” hours spent gaming per week, weekly gaming frequency, relative preference for strategy over action games) and cognitive abilities (short-term memory, working memory, and processing speed) on learning of two novel video games in 107 participants (aged 18–77 years). One video game was from the action genre, and the other was from the strategy genre. Hours spent gaming per week and working memory were found to specifically predict learning of the novel strategy video game, after controlling for the effects of age, gender, and action game learning. In contrast, self-identification as a “gamer” was the only specific significant predictor of action game learning, after controlling for the effects of age, gender, and strategy game learning. Age of the participant negatively impacted learning of both games; however, the pattern of the predictive relationships on both action and strategy game learning was not moderated by age. Importantly, a preference for the action versus the strategy game genre had no differential effects on learning of the two novel games, nor were there any gender differences in identification as a gamer or genre preference. Findings from this study suggest that while past gaming experience and cognition do appear to influence the learning of novel video games, these effects are selective to the game genre studied and are not as broad as the “learning to learn” model suggests.

## Introduction

Video games are not only an incredibly prevalent medium of entertainment (Lenhart et al., 2008; Rideout et al., 2010); they have become a much-researched topic of investigation within the cognitive sciences. A large body of work investigating the cognitive profiles of video game players has established an advantage in perceptual and spatial attentional skills for gamers who play for 3 h or more per week, compared to novices who play video games for, at most, 1 h per week (for a meta-analysis, see Bediou et al., 2018). In response to these findings, numerous experimental studies have been conducted to investigate the possibility of video game training directly enhancing cognition in not only younger adults aged 18–30 years but also older adults, aged 60 years and above, who have impaired cognitive abilities (Powers et al., 2013; Simons et al., 2016). While many such intervention studies have demonstrated positive cognitive outcomes (e.g., Green and Bavelier, 2006; Basak et al., 2008, 2020; Green et al., 2010; Toril et al., 2014), others have failed to identify training-related gains to cognition (e.g., Boot et al., 2013; van Ravenzwaaij et al., 2014; Minear et al., 2016). Given these mixed findings, the efficacy of video game interventions remains highly contentious (Bisoglio et al., 2014; Simons et al., 2016). One factor that may explain these mixed findings of video game intervention studies is *game genre*.

While terms vary, games within the cognitive training literature are most commonly divided into the following *game genres*: “action games,” “strategy games,” and “casual games” (see Simons et al., 2016, for a review). These genres have been argued to be an unreliable categorization of video games, particularly in cases where a game demonstrates features that can be attributed to multiple different genres (Dale and Green, 2017). However, studies that explicitly compared action games and strategy games to other games have shown differential benefits to cognition in younger adults (Cohen et al., 2008; Glass et al., 2013; Oei and Patterson, 2013, 2014; Wu and Spence, 2013). Training on the action game genre, such as first-person shooter (FPS) games and racing video games, selectively improved measures of attention, when compared to training on non-action games (Cohen et al., 2008; Wu and Spence, 2013; Oei and Patterson, 2014). In contrast, training on the strategy game genre, such puzzle games and real-time strategy (RTS) games, selectively improved working memory (including verbal and spatial and executive functions, when compared to other non-strategy and action games (Glass et al., 2013; Oei and Patterson, 2013, 2014). These results on strategy games from younger adults are extended to older adults, where training on RTS games has shown improvements in working memory, executive functions, and reasoning abilities (Basak et al., 2008; Whitlock et al., 2012), with faster learning of the RTS game being associated with greater improvements in executive functions and working memory (Basak et al., 2008) and with larger fronto-parietal and cerebellar gray matter volumes (Basak et al., 2011). In addition to these differential effects on cognition from action versus strategy game genres, studies have also shown partially different cognitive profiles that underlie performance on action versus strategy games (Baniqued et al., 2013; Ray et al., 2017). Working memory, reasoning, and processing speed were more correlated to games with strong reasoning and memory components—common attributes of the strategy game genre. Games with attention and speed components—common attributes of the action game genre—were related to measures of perception and reasoning (Baniqued et al., 2013; Ray et al., 2017). Together, these results from training and cognitive profiling indicate that the coarse genre distinction of action versus strategy does have some reliability and validity.

The genre distinction is supported by a neuroimaging study, where tract-based spatial statistics (TBSS) analysis of diffusion tensor imaging (DTI) was conducted in novice video gamers of a broad age range (18–80 years). Different regions of white-matter connectivity predicted selective learning of action versus strategy genres. Increased connectivity between limbic brain regions (fornix–stria terminalis), that are argued to underlie emotional arousal (Ravaja et al., 2004, 2006, 2008), predicted specifically action video game learning, whereas increased connectivity between subcortical regions that subserve memory (left cingulum–hippocampus) specifically predicted strategy video game learning. The casual games used in this study were Sushi-Go-Around, which combined attributes of the strategy game genre, and Tank Attack 3D, a shooter game that combined attributes of action game genre. Sushi-Go-Around was also investigated and classified in Baniqued et al. (2013). In the current study, we therefore limit our investigation to these two games, given the reliability and validity of these games under their respective genres. We assessed complex skill learning in these two novel games in an adult life span sample of participants who had not played this game before, and related the learning measures to past gaming experience and cognition. Given the past findings that showed that games with attention and speed components, such as FPS and shooter games, are related to perception, and that strategy games are related to memory (Baniqued et al., 2013) and brain structures underlying memory processing (Ray et al., 2017), we hypothesize that there will be a greater positive relationship between strategy novel-game game learning and cognition, specially working memory, compared to the relationship between action game learning and cognition.

Alternatively, we may observe just a general positive relationship between complex skill learning and cognition but no differential relationships across the two games, especially when accounting for gaming experience. The “learning to learn” model (Bavelier et al., 2012; Green and Bavelier, 2012) posits that enhanced cognitive ability observed in habitual video game players is not due to specifically bolstered attentional or memory capacity but, rather, an acquired ability to learn novel tasks for which they have no prior experience. Bavelier and colleagues specifically argue that, due to the wide variety of cognitive demands presented by different types of video games and the propensity of habitual video game players to periodically acquire and play new games, habitual game players have developed strategies and propensities which allow them to learn the intricacies of a novel, never-before-played video game—and by extension, other tasks that they have no prior exposure to—more quickly than non-players.

From this perspective, we hypothesize that individuals who either play video games more frequently, or identify themselves as gamers, or play for longer duration would demonstrate an advantage in novel game learning, irrespective of the gaming genre. To date, this hypothesis has not been tested with respect to novel game learning. However, one study observed no difference in learning rates on a serial reaction time task between habitual action video game players and non-gamers (Morin-Moncet et al., 2016), which runs counter to the primary prediction of the “learning to learn” model. Moreover, there is paucity of studies reporting any correlation between duration or frequency of game play and cognition. To date, only one study has reported scaled benefits to motor control and visual perception related to self-reported hours of weekly game play (Rupp et al., 2019).

In the current study, not only will we evaluate the relationships between novel game learning across the two genres and cognition; we will also determine if these relationships are mitigated by game experience, such as gaming frequency or lengths of time spent on gaming or self-identification as a gamer. We hypothesize that gaming frequency and gaming duration will be significantly correlated with learning of both new games, based on the “learning to learn” hypothesis. However, it is not known whether there are any specific, differential effects of past game experiences on the two different games under investigation. If these game experience variables predict similarly the learning of the two games, we will find support for the “learning to learn” model, whereas differential predictors of these two different genres of games would suggest that the influence of past experience on novel game learning is more nuanced than the “learning to learn” model suggests.

Additionally, we will also evaluate at post-learning participant’s preference ratings of the two games played. It is possible that the speed with which we learn a game may influence our preference for that game compared to the other, and this preference may influence cognition–learning relationships. However, if no relationships between game preference and game learning are found, then the specific effects of cognition and game experience on learning of the two games can be considered to be independent of individual differences in game preference.

An important aspect of the current study is the use of a life span sample. Most studies on video games experience and cognition are restricted to younger adults and therefore cannot be generalized to other age groups. Given the prevalence of video games as a cognitive intervention tool in older adults (for meta-analyses, see Lampit et al., 2014; Toril et al., 2014; Basak et al., 2020), it is important that we understand these relationships from an adult life span perspective. However, not all types of cognitive interventions have similar beneficial effects on cognition, especially on *far* cognitive abilities that were not trained. In a recent meta-analysis, effects of cognitive training from randomized control trials on healthy older adults and older adults with mild cognitive impairments (MCIs) were evaluated (Basak et al., 2020). In particular, cognitive training targeting a single cognitive component was compared to those training multiple cognitive components. Single cognitive component training studies were further separated into following modules: speed, executive functions (including switching, inhibition, and working memory updating), reasoning, and memory (including memory training strategies). Only executive functions (including working memory) training and memory training had significant effects on far cognitive abilities, measured by tasks that are unrelated to the training task(s). Therefore, it is possible that certain genres of video games, depending on what cognitive components they rely upon, may be more effective as cognitive intervention tools, particularly in middle and late adulthood. The current study, by engaging a life span approach that incorporates not only young adults but also older adults as participants, is poised to investigate if speed and working memory are correlated differently to action and strategy game learning and whether these effects are moderated by age. This research is significant because it has the potential to inform on what game genres may be the best for cognitive interventions, tailored to a participant’s cognition, past gaming experience, age, and game preferences.

## Materials and Methods

### Participants

One hundred and seven adults (40 females; *M*_*Age*_ = 34.79, SD_*Age*_ = 20.59; aged 18–77) participated in this study. Fifty-three participants were recruited from the University of Texas at Dallas and received class credit for their participation. The remaining 54 participants were recruited from Dallas and its neighboring communities and were paid $10/h for their participation. Inclusion criteria were high school education (or more), normal or corrected-to-normal vision (visual acuity >20/30), and no reported history of major medical or psychological illnesses (e.g., visual neglect, epilepsy, heart attacks, etc.). Participants aged 55 and older were required to score 26 or above in the Mini-Mental State Examination (MMSE 2nd Edition; Folstein et al., 2010), an indicator of normal cognitive health.

### Ethical Approval and Informed Consent

The study was approved by the Institutional Review Board of the University of Texas at Dallas and performed in accordance with the ethical standards for research outlined by that review board. Written and informed consent was collected from all participants included in this study prior to any testing procedures.

### Procedures

In this 2 h study, after informed consent, participants underwent cognitive assessments of processing speed (a measure of attention), short-term memory, and working memory. Then the participants played two novel online video games for which they had no reported prior experience. These games were played in succession for 40 min each (80 min total). The order of the games played was counterbalanced between the participants. Finally, participants answered a survey of their past gaming experience and their preferences on the two games played.

### Cognitive Measures

Cognitive tests administered included the following: forward digit span (*FSpan*), a measure of short-term memory that requires temporary storage of digits for a very short period of time (*WAIS-R: Wechsler Adult Intelligence Scale—Revised*; Wechsler, 1981); backward digit span (*BSpan*), a measure of working memory that requires, in addition to storage, manipulation of the digits in mental workspace (*WAIS-R*; Wechsler, 1981); and the Symbol–Digit Substitution Test (*SDST*, taken from the *MMSE 2nd Edition*), a measure of processing speed (Folstein et al., 2010).

### Gaming Genres and Calculation of Learning Composites

The two video games played were Tank Attack 3D, an action game, and Sushi-Go-Round, a strategy game, on miniclip.com, a website which hosts free casual games spanning multiple genres (Baniqued et al., 2013). Games from minilip.com were utilized due to their ease of access and short individual play duration, which allowed for multiple iterations of the game to be played in a relatively short time period and facilitated the assessment of game learning quickly. Additionally, both games feature adaptive difficulty, introducing new and more challenging mechanics in response to high performance by the player, which allowed us to assess the degree to which our participants learned these mechanics by proxy of progression through each game. Lastly, both of these selected games conform to the “action” and “strategy” genres as defined by the video game cognition literature, thereby avoiding issues of interpretation presented by more complex, longer-playing video games that involve multiple genre features (Dale and Green, 2017).

**FIGURE 1:**
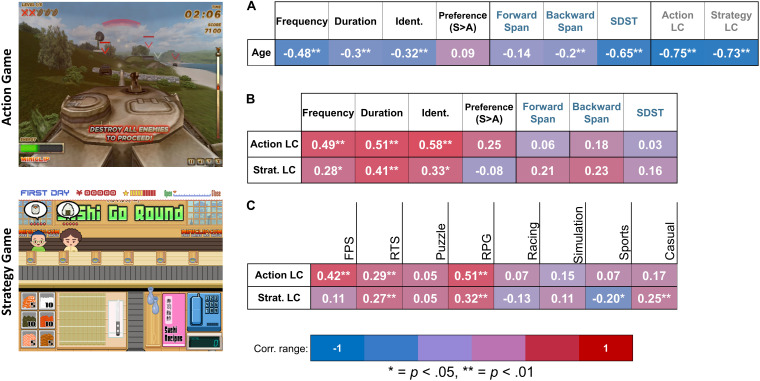
Results of correlation analyses performed. Panel **(A)** depicts Pearson’s correlations between age and our variables of interest. Panel **(B)** depicts partial correlations between Action and Strategy LCs (learning composites) and our cognitive and game habit variables of interest, controlling for age and gender. Panel **(C)** depicts partial correlation between Action and Strategy LCs and participant reports of experience with eight specific genres of games, controlling for age and gender. Ident. = identification as a “gamer.”

Tank Attack 3D (Figure 1) was selected as an exemplar action game due to its strong perceptual and attentional demands, coupled with relatively minimal resource management and decision-making requirements (Baniqued et al., 2013; Ray et al., 2017). In Tank Attack 3D, players are required to operate a tank through a war area to destroy enemy radars, tanks, and bases. To complete the mission successfully, players were required to arrive at their home base with remaining energy before the timer ran out. In addition to the time awareness required for successful play, players had to react to fast-moving stimuli and keep track of multiple items, such as the number of radars destroyed and life energy left. Additionally, Tank Attack 3D periodically introduces new types of enemies in response to player victories, forcing the player to adapt to those new challenges to succeed in later missions.

Sushi-Go-Round (Figure 1) was selected as an exemplar strategy game due to its strong emphasis on resource management, coordination between multiple information units and tasks, rapid reasoning, and decision making (Baniqued et al., 2013; Ray et al., 2017). In Sushi-Go-Round, players are required to serve a variety of sushi preparations to a continuous stream of customers while simultaneously monitoring customer happiness, order times, remaining ingredients, and monetary incomes and expenses. To complete a level successfully, players were required to accumulate a certain monetary value by serving customers the requested dishes, while avoiding making the customers wait too long, serving the customer an incorrect dish, or making errors in dish preparation. Additionally, Sushi-Go-Round periodically introduces new and more complex recipes to the menu in response to player success, forcing players to continuously learn new recipes to succeed in later levels.

Participants were asked to play each game continuously for 40 min, and the play order was counterbalanced across participants. A round of either game lasted for about 7 min. The games also become adaptively more difficult, indexed by the game level, based on the participant’s performance. In cases where a round was in progress after 40 min had elapsed, participants were asked to finish that round and then cease play.

A *learning composite (LC)* measure was calculated for each game for each participant based on two outcome measures: *highest level* reached in each game at the end of 40 min and the *learning rate*. *Learning rate* was calculated as the slope of a logarithmic function fitted to each participant’s scores (higher scores indicate higher success in game play). A steeper slope implies faster learning and greater improvement (Basak et al., 2011; Ray et al., 2017). *Highest level reached* reflected the difficulty tier reached in each game and was reported as “day” for Sushi-Go-Round and “mission” for Tank Attack 3D. These two measures (*learning rate* and *highest level reached)* were first standardized (z-scored) and then averaged to create the *LC*. We utilized a composite measure to assess learning rates as a method of insulating against fatigue effects: the score output of both Sushi-Go Round and Tank Attack 3D fluctuates with the participant’s performance and can drop over time if the participant becomes fatigued or less engaged with the task. *Highest level*, conversely, can never fall as a result of poor performance in these games and, as discussed above, is a proxy of the participant’s learning of the mechanics of each game. Constructing these measures allowed us to examine two different metrics of learning on each game, while lessening the overall measure’s susceptibility to fatigue effects, and is consistent with our approach in previous published research which assessed learning rates on these two games or similar games (Boot et al., 2010; Basak et al., 2011; Ray et al., 2017).

### Past Gaming Habits and Preferences Survey

Participants completed a survey of gaming habits and preferences adapted from that used by Basak et al. (2008, 2011) and Boot et al. (2008) to assess the level of video game skill (i.e., to identify expert or novice players). This adapted questionnaire addressed the participants’ typical video game habits on a weekly basis, as is the standard methodology used in the game cognition field to differentiate between non-players and players of various skill levels (Green and Bavelier, 2006; Feng et al., 2007; Basak et al., 2008, 2011; Boot et al., 2008; McDermott et al., 2014).

Unless otherwise noted, responses were recorded on a five-point Likert scale for the questions in the survey. Individuals rated how frequently they played different genres and formats of video games from “never” to “often”; ratings were collapsed across five common game formats (personal computer game, game arcade, home gaming console, internet gaming website, phone/tablet game) to determine an individual’s gaming *Frequency*. Experience with specific action game subgenres (FPS, racing, and simulation); specific strategy game subgenres [RTS, role-playing games (RPGs), simulation, and puzzle]; and casual games was assessed on the same scale. *Identification* as a gamer was rated on a five-point Likert scale of “do not identify at all” to “strongly identifies.”

*Gaming duration* was measured via a numeric report of estimated weekly hours spent gaming over the past month. These variables (*Frequency, Duration, Identification*) constitute an individual’s game habits for the purpose of this study.

In addition to these questions, participants also rated their level of enjoyment of playing the two games used in this study (1 = did not enjoy, 5 = highly enjoyable). The enjoyment rating for Tank Attack 3D was subtracted from that of Sushi-Go-Round to yield the game *Preference* score; a positive score indicates greater preference for Sushi-Go-Round, whereas negative score indicates greater preference for Tank Attack 3D.

### Justification of Statistical Power

We have sufficient power to not only examine correlations between two variables but also conduct multiple regressions required to evaluate the cognition–learning, experience–learning, and cognition–experience–learning relationships. All estimations for power analyses were conducted using G^∗^power3.1 (Faul et al., 2009). Our sample size of 107 participants provides us with a power greater than 0.95 to detect an effect size of 0.5 in a regression analysis with only one predictor, and a power greater than 0.92 to detect the same effect size in a multiple regression analysis with three predictors (e.g., three cognitive variables as predictors of game learning). We also have a power greater than 0.8 to detect an effect size of 0.5 in a multiple regression analysis with all seven regressors of interest in this study, that is, three cognitive variables (*FSpan, BSpan, SDST*), three game habit variables (*Identification, Duration, Frequency*), and one game preference variable.

## Results

### Descriptive Statistics of Cognitive and Game Habit Variables

Table 1 reports means and standard deviations of responses to the game habit variables (*Frequency, Duration, Identification)* as well as means and standard deviations of the cognitive assessments administered (*FSpan, BSpan, SDST*) and of game *Preference*. Notably, the primary *Preference* measure (relative preference of Sushi-Go-Round over Tank Attack, *S-A*) indicated that participants preferred Tank Attack 3D (action game) over Sushi-Go-Round (strategy game), mean *S-A* = −0.29, SD = 1.72. A paired-samples t-test comparing reported preference for each of these games found this difference to be significant, *t*(106) = 2.26, *p* = 0.03.

**TABLE 1:** Means and standard deviations of behavioral and cognitive measures (left), and reported frequency of experience with specific game genres (right).

**Primary measure**	**Mean (SD)**	**Genre experience**	**Mean (SD)**
Frequency	2.14 (0.78)	FPS game	1.48 (0.75)
Duration (h/week)	2.01 (1.53)	RTS game	1.58 (0.8)
Identification	2.31 (1.33)	Puzzle game	1.81 (0.81)
Preference (*S > A*)	−0.29 (1.72)	RPG	1.82 (1.18)
FSpan	6.87 (1.31)	Racing game	1.73 (0.95)
BSpan	5.3 (1.17)	Simulation game	1.59 (0.85)
SDST	22.98 (5.12)	Sports game	1.55 (1.11)
		Casual game	1.94 (1)

*FSpan, forward digit span; BSpan, backward digit span; SDST, Symbol–Digit Substitution Test; FPS, first-person shooter; RTS, real-time strategy; RPG, role-playing game.*

### Relationships Between Cognition, Game Habits, Game Preference, and Novel Game Learning

**FIGURE 2:**
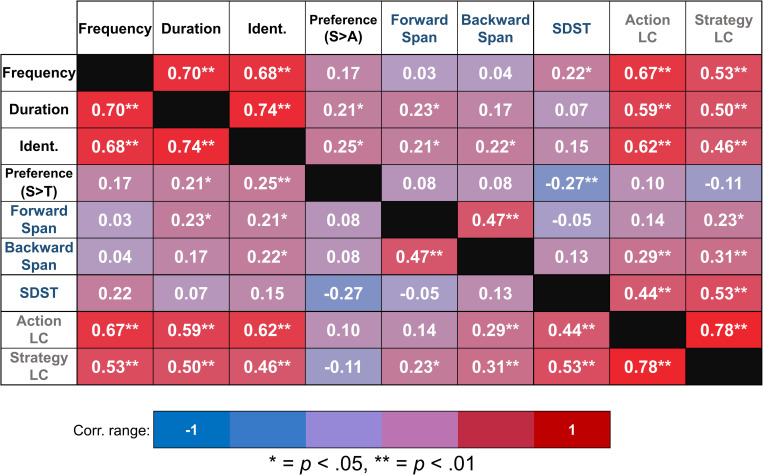
Results of Pearson’s correlations between our variables of interest.

We ran a series of bivariate correlations, using Pearson’s method, between the variables of game habits, cognition, and novel game learning, as well as *Preference*, to establish baseline relationships between these measures. The results of these correlations are presented in Figure 2. The game habit variables (*Frequency, Duration*, and *Identification*) were significantly inter-correlated: *Frequency–Duration, r*(106) = 0.7, *p* < 0.01; *Frequency–Identification, r*(106) = 0.68, *p* < 0.01; *Duration–Identification, r*(106) = 0.74, *p* < 0.01. Similarly, both *Action LC* and *Strategy LC* were significantly correlated, *r*(106) = 0.78, *p* < 0.01. These bivariate correlations suggest that the multiple measures of the two constructs, *game habit* and *novel game learning*, were strongly inter-correlated and were reliable measures of these constructs. For cognition, *FSpan* and *BSpan* were significantly correlated, *r*(106) = 0.47, *p* < 0.01, but neither was significantly correlated with *SDST*, [*FSpan r*(106) = −0.06, *p* = 0.6; *BSpan r*(106) = 0.13, *p* = 0.21].

Regarding the habit–learning relationship, all measures of game experience (*Frequency*, *Duration*, and *Identification*) were significantly and positively correlated with both Action LC [*Frequency, r*(106) = 0.67, *p* < 0.01; *Duration, r*(106) = 0.59, *p* < 0.01; *Identification, r*(106) = 0.62, *p* < 0.01] and Strategy LC [*Frequency, r*(106) = 0.53, *p* < 0.01; *Duration, r*(106) = 0.5, *p* < 0.01; *Identification, r*(106) = 0.46, *p* < 0.01]. Regarding habit–cognition relationships, *Duration* was significantly correlated with *FSpan*, *r*(108) = 0.23, *p* = 0.03, as was *Identification*, *r*(106) = 0.21, *p* = 0.04. *Identification* was additionally significantly correlated with b *BSpan*, *r*(106) = 0.22, *p* = 0.03. These results suggest that greater gaming experience was related to better learning of novel games, better working memory, and to a lesser degree, better short-term memory.

*Preference* was significantly and positively related to both *Duration*, *r*(106) = 0.21, *p* = 0.03, and *Identification*, *r*(106) = 0.25, *p* = 0.01, but not with *Frequency*, *r*(106) = 0.17, *p* = 0.08, indicating that participants who reported a higher preference for the action game than the strategy game tended to also report, on average, more hours of gameplay per week and more strongly identified as “gamers.” *Preference* was not related to either of the two memory span measures [*FSpan*, *r*(106) = 0.08, *p* = 0.48; *BSpan*, *r*(106) = 0.08, *p* = 0.43], nor to either LC [*Action LC*, *r*(106) = 0.1, *p* = 0.29; *Strategy LC*, *r*(106) = −0.11, *p* = 0.26]. *SDST* was, however, significantly but negatively correlated with preference of the action game over the strategy game, *r*(106) = −0.27, *p* = 0.01. This result suggests that individual differences in greater preference for the strategy game over the action game was related to better performance on SDST (a measure of processing speed), although preference for one type of game over another was not impacted by individual differences in memory spans, either *FSpan* or *BSpan*. The results from the effects of *Preference* for a specific game on learning of the two games are reported in detail in section “Additional Analyses: Effect of Game Preference on Novel Game Learning.”

### Age and Gender as Predictors of Novel Game Learning

In order to investigate the potential influence of demographic variables on novel game learning, we first analyzed the relationships of participants’ age and gender with the other variables (game habits, preference, cognition, and learning).

Bivariate correlations using Pearson’s method indicated that age was inversely correlated with *Frequency*, *r*(106) = −0.48, *p* < 0.01, *Duration*, *r*(106) = −0.3, *p* < 0.01, and *Identification*, *r*(106) = −0.32, *p* < 0.01, as well as LCs of both action, *r*(106) = 0.75, *p* < 0.01, and strategy, *r*(106) = −0.73, *p* < 0.01, games. Age was not significantly correlated with *Preference*, *r*(106) = 0.09, *p* = 0.36. For cognition, age was inversely correlated with working memory capacity (*BSpan*), *r*(106) = −0.2, *p* = 0.06, and processing speed (*SDST*), *r*(106) = −0.65, *p* < 0.01, but not with STM (*FSpan*), *r*(106) = −0.14, *p* = 0.19 (Figure 1A).

**FIGURE 3:**
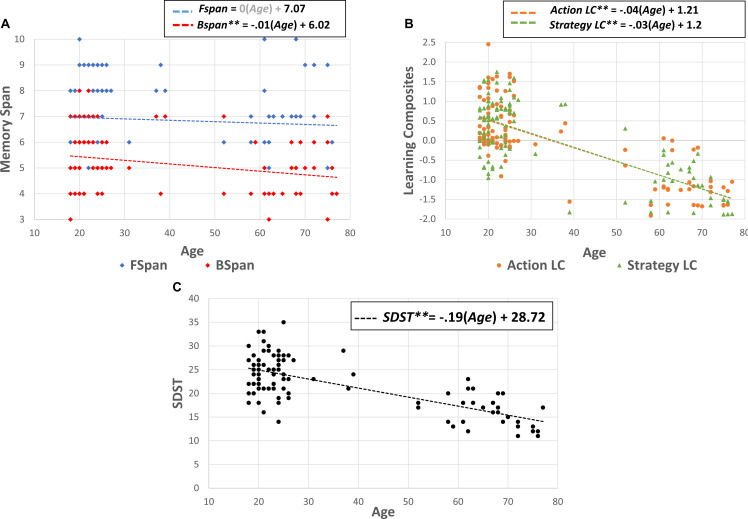
Scatterplots of age–cognition and age–learning relationships, with linear trendiness plotted. Panel **(A)** depicts the relationship between age and the forward (*FSpan*) and backward (*BSpan*) memory span measures. Panel **(B)** depicts the relationship between age and the Action LC and Strategy LC. Panel **(C)** depicts the relationship between age and the Symbol–Digit Substitution Test (SDST).

Considering the asymmetry of the age distribution of our sample, we next fitted linear models to each of the age-to-habit, age-to-cognition, and age-to-learning relationships to establish if these relationships are linear and, therefore, can be subjected to linear regressions in subsequent analyses. Regarding *habit* variables, the relationship between age and *Frequency*, *F*(1/105) = 31.61, *p* < 0.01, *R*^2^ = 0.23, age and *Duration*, *F*(1/105) = 13.8, *p* < 0.01, *R*^2^ = 0.11, and age and *Identification, F*(1/105) = 14.14, *p* < 0.01, *R*^2^ = 0.12, was significantly linear. Age and *Preference* demonstrated no such relationship, linear or nonlinear, *F*(1/105) = 0.87, *p* = 0.35, *R*^2^ = 0.01. Regarding *Cognition*, age and *BSpan*, *F*(1/105) = 7.03, *p* = 0.01, *R*^2^ = 0.06, and age and *SDST*, *F*(1/105) = 117.67, *p* < 0.01, *R*^2^ = 0.52, demonstrated significant linearity (Figures 3A,C), but age and *FSpan* displayed no significant relationship (Figure 3A), *F*(1/105) = 0.71, *p* = 0.4, *R*^2^ = 0.01. Age also demonstrated a linear relationship with both the Action LC, *F*(1/105) = 138.17, *p* < 0.01, *R*^2^ = 0.57, and the Strategy LC, *F*(1/105) = 124.11, *p* < 0.01, *R*^2^ = 0.54 (Figure 3B).

Additionally, we ran a series of independent sample *T*-tests comparing male and female participants on habits, preference, learning, and cognitive variables. No significant gender differences were observed in these comparisons (Table 2).

**TABLE 2:** Results of *T*-tests comparing male and female participants on variables of game habits cognition and learning.

**Measure**	**Mean (SD)_*M*_**	**Mean (SD)_*F*_**	***t* (*df*)**	***P***
Frequency	2.1 (0.82)	2.2 (0.72)	0.62 (105)	0.54
Duration (h/week)	2.02 (1.49)	2.17 (1.61)	0.51 (105)	0.61
Identification	2.24 (1.34)	2.41 (1.32)	0.67 (105)	0.51
Preference (*S > A*)	−0.27 (1.95)	−0.32 (1.29)	−0.14 (105)	0.89
FSpan	6.89 (1.4)	6.83 (1.18)	−0.23 (105)	0.82
BSpan	5.21 (1.13)	5.46 (1.25)	0.98 (105)	0.33
SDST	23.56 (5.2)	22.06 (4.9)	−1.39 (105)	0.17
Action LC	−0.07 (0.95)	0.14 (0.93)	1.09 (105)	0.28
Strategy LC	−0.04 (0.96)	0.07 (0.98)	0.6 (105)	0.55

*LC, learning composite.*

### Gaming Habits and Cognition as Predictors of Novel Game Learning

The primary intent of this study is to examine the relationship between past gaming habits, cognitive abilities, game preference, and the learning of novel games, across life span, irrespective of age and gender differences. Although these variables are correlated (see section “Relationships Between Cognition, Game Habits, Game Preference, and Novel Game Learning”), it not clear to what extent these relationships are driven by individual differences in age. Therefore, we conducted correlations between these variables, after controlling for age and gender. Action LC was still significantly correlated with *Frequency*, *r*(104) = 0.49, *p* < 0.01, *Duration*, *r*(104) = 0.52, *p* < 0.01, and *Identification*, *r*(104) = 0.58, *p* < 0.01. Likewise, Strategy LC was correlated with *Frequency*, *r*(106) = 0.28, *p* = 0.04, *Duration*, *r*(104) = 0.41, *p* < 0.01, and *Identification*, *r*(104) = 0.33, *p* < 0.02 (Figure 1B). *Preference* was now marginally correlated with Action LC, *r*(104) = 0.25, *p* = 0.07, but not with Strategy LC, *r*(104) = −0.08, *p* = 0.59, after controlling for age and gender. None of the cognitive variables examined were found to be correlated with either Action LC [*FSpan*: *r*(104) = 0.09, *p* = 0.61; *BSpan*: *r*(104) = 0.18, *p* = 0.29; *SDST*: *r*(104) = 0.03, *p* = 0.87] or Strategy LC [*FSpan*: *r*(104) = 0.21, *p* = 0.22; *BSpan*: *r*(104) = 0.23, *p* = 0.16; *SDST*: *r*(104) = 0.15, *p* = 0.35], after controlling for age and gender.

To determine if the relationships between novel game learning and game habits, preference, and cognition are linear, we fit linear models to these relationships. The linear relationships between Action LC and *Frequency*, *F*(1/105) = 70.09, *p* < 0.01, *R*^2^ = 0.4, *Duration*, *F*(1/105) = 63.92, *p* < 0.01, *R*^2^ = 0.38, *Identification*, *F*(1/105) = 63.59, *p* < 0.01. *R*^2^ = 0.37, *BSpan, F*(1/105) = 11, *p* < 0.01, *R*^2^ = 0.09, and *SDST*, *F*(1/105) = 47.7, *p* < 0.01, *R*^2^ = 0.31, were significant. The linear relationships between Action LC and *Preference*, *F*(1/105), *p* = 0.33, *R*^2^ = 0.01, and between Action LC and *FSpan*, *F*(1/105) = 2.33, *p* = 0.12, *R*^2^ = 0.02, were not significant. The relationships between the Sushi LC and variables of game habit and cognition had similar patterns [*Frequency: F*(1/105) = 41.71, *p* < 0.01, *R*^2^ = 0.28; *Duration: F*(1/105) = 40.91, *p* < 0.01, *R*^2^ = 0.28; *Identification: F*(1/105) = 30.24, *p* < 0.01, *R*^2^ = 0.22; *BSpan: F*(1/105) = 10.8, *p* < 0.01, *R*^2^ = 0.09; *SDST: F*(1/105) = 62.36, *p* < 0.01, *R*^2^ = 0.37; *Preference: F*(1/105) = 1.32, *p* = 0.25, *R*^2^ = 0.01; *FSpan: F*(1/105) = 3.18, *p* = 0.08, *R*^2^ = 0.03].

These results above demonstrate that measures of novel game learning in both genres are related individually to measures of game experience, over and beyond the effects of age and gender. However, they do not reflect the potentially interrelated effects of our variables of interest on novel game learning. To investigate this possibility, we next performed a series of multiple regressions to examine the combined effect of the collected gaming experience and cognition on the game LCs. Two sets of stepwise multiple regressions were conducted, one set using Action LC and another set using Strategy LC as the dependent variable. In both stepwise multiple regressions, age and gender were entered as control variables in the first step. In the second, game experience variables (*Frequency, Duration, Identification*), and cognitive variables (*FSpan, BSpan, SDST*) were entered, which resulted in a handful of significant predictors. The results of these analyses are presented in Table 3. The final regression model for Action LC, *R*^2^ = 0.67, *F*(4,102) = 41.9, *p* < 0.01, included *Identification*, β = 0.15, *t*(102) = 2.78, *p* < 0.01, and *Duration*, β = 0.11, *t*(102) = 2.46, *p* = 0.02, as significant predictors. The final regression model for Strategy LC, *R*^2^ = 0.57 *F*(4,102) = 27.67, *p* < 0.01, included *Duration*, β = 0.21, *t*(102) = 4.79, *p* < 0.01, and *BSpan*, β = 0.15 *t*(102) = 2.58, *p* = 0.01, as significant predictors.

**TABLE 3:** Results of stepwise regression across 3 steps.

	**Action LC Models**				**Strategy LC Models**				
**Model**	***R*^2^**	***ΔR^2^***	***F***	***p***	**Model**	***R*^2^**	***ΔR^2^***	***F***	***p***

1) Age + Gender	0.57	−	69.3	**<0.01**	1) Age + Gender	0.53	−	58.82	**<0.01**
2) + Identification	0.73	0.15	90.89	**<0.01**	2) + Duration	0.62	0.09	56.66	**<0.01**
3) + Duration	0.75	0.02	74.6	**<0.01**	3) + BSpan	0.64	0.02	45.45	**<0.01**

	**Regression Model from Step 3**					**Regression Model from Step 3**			
**Factors**	***β***	***t***	***p***		**Factors**	***β***	***t***	***p***	

Age	−0.03	−10.9	**<0.01**		Age	–0.03	–9.13	**<0.01**	
Gender	−0.12	−1.25	0.2		Gender	0.02	0.15	0.89	
Identification	0.19	3.47	**<0.01**		Duration	0.19	4.8	**<0.01**	
Duration	0.13	2.79	**0.01**		BSpan	0.12	2.25	**0.03**	

*Details of final regression model from step 3 are provided. Bold values indicate p < 0.05.*

### How Specific Are These Effects?

The above multiple regressions indicate that gaming duration is a common predictor of both action and strategy learning, but this result can be driven by the inter-correlation between the Action and Strategy LCs. A correlation analysis indeed demonstrated that Action LC and Strategy LC were significantly related, *r*(106) = 0.78, *p* < 0.01. To account for this inter-correlation, two sets of stepwise multiple regression analyses were conducted, such that the effects of gaming experience and cognition on either game learning could be determined, over and beyond the effects of the other game learning.

As in previous regressions, the first step included age and gender, and the second step included the other game LC (that is, Strategy LC when Action LC was the dependent variable, and Action LC when Strategy LC was the dependent variable). For the third step, only the significant predictors of earlier regression analyses (see Table 3) for a specific game learning were used to determine their specificity on that game learning. For example, for Action LC, *Identification* and *Duration* were entered in the third step. For Strategy LC, *BSpan* and *Duration* were entered in the third step. The results of these analyses are reported in Table 4.

**TABLE 4:** Results from follow-up regression models, after controlling for opposite game learning.

	**Action LC Models**					**Strategy LC Models**			
**Model**	***R*^2^**	***ΔR^2^***	***F***	***p***	**Model**	***R*^2^**	***ΔR^2^***	***F***	***p***
1) Age + Gender	0.57	−	69.3	**<0.01**	1) Age + Gender	0.53	−	58.82	**<0.01**
2) + Strategy LC	0.68	0.11	73.36	**<0.01**	2) + Action LC	0.65	0.12	64.06	**<0.01**
3) + Add. Predictors	0.78	0.10	69.55	**<0.01**	3) + Add. Predictors	0.68	0.03	42.3	**<0.01**
									
	**Regression Model from Step 3**				**Regression Model from Step 3**		
**Factors**	***β***	***t***	***p***	**Factors**	***β***	***t***	***p***
Age	−0.02	−5.95	**<0.01**	Age	–0.02	–4.23	**<0.01**
Gender	−0.12	−1.35	0.18	Gender	0.06	0.56	0.58
Strategy LC	0.28	3.65	**<0.01**	Tank LC	0.37	3.36	**<0.01**
Identification	0.18	3.45	**<0.01**	Duration	0.10	2.22	**<0.03**
Duration	0.08	1.78	*0.08*	BSpan	0.09	1.77	*0.08*

*Additional predictors in Step 3 were significant predictors from the previous step-wise regressions described in Table 3. Bold values indicate p ¡ 0.05; Italicized values indicate p < 0.10.*

*Identification* remained a significant predictor of *Action LC* even after accounting for Strategy LC, β = 0.16, *t*(101) = 3.16, *p* < 0.01, but *Duration* was no more a significant predictor, β = 0.05, *t*(101) = 1.13, *p* = 0.26. However, *Duration* remained a significant predictor of Strategy LC even after accounting for Action LC, β = 0.14, *t*(101) = 2.83, *p* < 0.01, with *BSpan* still being a marginally significant predictor of Strategy LC, β = 0.11, *t*(101) = 1.94, *p* = 0.056.

### Are These Predictors Stable Across the Life Span?

So far, our analyses have focused on the effects of game habits, preference, and cognition on novel game learning, irrespective of age. However, it is plausible that the pattern of these relationships varies across the adult life span. We therefore performed moderator analyses using multiple regressions to account for the effects of age on these relationships. Gender was introduced in the first step of these multiple regressions. Age and the significant predictors demonstrated previously (*Identification* and *Duration* for the Action LC, *Duration* and *BSpan* for the Strategy LC) were entered in the second step. The third step included the moderation terms that accounted for the interaction between age and the two variables, which were entered in the second step. The results of these analyses are reported in Table 5.

**TABLE 5:** Results of moderator analyses.

	**Action LC Models**					**Strategy LC Models**			
**Model**	***R*^2^**	***ΔR^2^***	***F***	***p***	**Model**	***R*^2^**	***ΔR^2^***	***F***	***p***
1) Gender	0.01	−	1.2	0.28	1) Gender	0.01	−	0.09	0.77
2) + Age & Add. Preds.	0.75	0.74	74.59	**<0.01**	2) + Age & Add. Preds.	0.64	0.63	45.45	**<0.01**
3) + Moderators	0.76	0.01	51.34	**<0.01**	3) + Moderators	0.65	0.01	30.36	**<0.01**
									
	**Regression Model from Step 3**				**Regression Model from Step 3**		
**Factors**	***β***	***t***	***p***	**Factors**	***β***	***t***	***p***
Gender	−0.11	−1.12	0.27	Gender	0.02	0.18	0.85
Age	−0.03	−9.17	**<0.01**	Age	–0.02	–6.34	**<0.01**
Identification	0.19	3.48	**<0.01**	Duration	0.21	4.82	**<0.01**
Duration	0.15	2.98	**<0.01**	BSpan	0.13	2.48	**0.02**
Age * Identification	−0.14	−1.89	*0.09*	Age * Duration	0.09	1.07	0.29
Age * Duration	0.16	1.74	*0.06*	Age * BSpan	0.04	0.69	0.49

*Bold values indicate p < 0.05; Italicized values indicate p < 0.10.*

While age was demonstrated to be a significant predictor of both Action LC, β = −0.03, *t*(100) = −6.61, *p* < 0.01, and Strategy LC, β = −0.03, *t*(100) = −0.4.18, *p* = 0.01, none of the interaction terms with *Age* demonstrated significance in either analyses, and neither of the regression models in the third step demonstrably improved model fit compared to the previous step, indicating that age is not a meaningful moderator of the previously observed relationships.

### Game Experience With Specific Genres

In addition to the general game experience/habits data collected, we also gathered information from each participant regarding their frequency of play with eight specific genres of video games. We examined the potential relationship between experience on a specific game genre and novel game learning through correlation analyses that compared self-rated frequency of playing of these genres with Action and Strategy LCs. These correlations were controlled for individual differences in age and gender. The results are displayed in Figure 1C.

Action LC was found to be positively correlated with frequent experience with FPS games, *r*(103) = 0.42, *p* < 0.01, RTS games, *r*(103) = 0.29, *p* < 0.01, and RPGs, *r*(103) = 0.51, *p* < 0.01. Strategy LC was positively correlated with frequent experience with RPG, *r*(103) = 0.32, *p* = 0.01, and casual games, *r*(103) = 0.25, *p* = 0.01 Additionally, Strategy LC was inversely correlated with frequent experience with sports video games, *r*(103) = −0.2, *p* = 0.04.

### Additional Analyses: Effect of Game Preference on Novel Game Learning

To further examine the impact of game preference on novel game learning, we next divided our participants into subgroups based on self-reported preference for the two games utilized in this task. Subjects were divided into three groups based on their reported enjoyment of Tank Attack 3D and Sushi-Go-Round. The *Prefer Tank* group (*n* = 34, 10 females, *M*_*Age*_ = 29.79, SD_*Age*_ = 18.14) reported higher enjoyment of Tank Attack 3D compared to Sushi-go-Round. The *Prefer Sushi* (*n* = 46, 17 females, *M*_*Age*_ = 25.89, SD_*Age*_ = 11.53) group reported higher enjoyment of Sushi-go-Round than Tank Attack 3D. The *No Preference* (*n* = 27, 13 females, *M*_*Age*_ = 55.79, SD_*Age*_ = 20.96) group reported identical levels of enjoyment of both games.

We first assessed how these preference groups differed in regard to the game LCs. A one-way ANOVA demonstrated that preference group had a significant effect on *Tank LC*, *F*(2,104) = 29.86, *p* < 0.01. Post hoc comparisons using the Sidak method demonstrated that *Tank LC* was significantly higher for the *Prefer Tank* group compared to the *No Preference* group (*p* < 0.01) but did not differ significantly from the *Prefer Sushi* group (*p* = 0.36). A second one-way ANOVA demonstrated that preference group had a significant effect on *Sushi LC* as well, *F*(2,104) = 20.71, *p* < 0.01. Post hoc comparisons using the Sidak method demonstrated that *Sushi LC* was significantly higher for the *Prefer Sushi* group compared to the *No Preference* group (*p* < 0.01) but did not differ significantly from the *Prefer Tank* group (*p* = 0.26). These results indicate that individuals with a preference for either game demonstrated greater LCs on both the action and strategy games utilized. Therefore, greater preference for a specific game was not related to better learning of that game.

Considering the apparent disparity in age between the three preference groups as reported above, we next compared participant age across these three groups. A one-way ANOVA indeed demonstrated a significant difference in age between the three groups, *F*(2,104) = 31.06, *p* < 0.01. Post hoc comparisons using the Sidak method demonstrated that the *No Preference* group was significantly older than both the *Prefer Tank* group (*p* < 0.01) and the *Prefer Sushi* group (*p* < 0.01), but there was no significant age difference between the *Prefer Tank and Prefer Sushi* groups (*p* = 0.65). These results suggest that the differences in relative learning rates between the preference groups and the no-preference group are possibly driven by participant age.

## Discussion

The purpose of this study was to examine the relationship between gaming experience, cognition, and the learning of novel games, particularly any specific patterns of relationships for the two different video game genres, action and strategy, which have been most predominant as cognitive training tools in past research (Oei and Patterson, 2014; Simons et al., 2016; Ray et al., 2017). Our results demonstrated a strong correlation between learning of both genres of video games and the gaming experience (*Frequency, Duration, Identification*), such that longer hours spent gaming per week (*Duration*), greater gameplay frequency, and greater extant of self-identification as a “gamer” (*Identification*) were related to faster learning of both action and strategy video games. These results seem to support the “learning to learn” framework, which predicts general enhancement of novel game learning as a result of previous game experience (Bavelier et al., 2012; Green and Bavelier, 2012). However, after controlling for the learning of the other game, *Duration* demonstrated a significant relationship only with the strategy game learning, not with action game learning. This result suggests that the observed positive relationship between *Duration* and action game learning may be spurious, contrary to the “learning to learn” model’s prediction of broad transfer to novel task learning. Both the present study as well as past research which has utilized these specific games (Baniqued et al., 2013; Ray et al., 2017) have demonstrated that Tank Attack 3D and Sushi-Go-Round have distinct patterns of cognitive correlates, which may have factored into the differential effects of game habits on the learning of these two games that we observed. It is conceivable that past game experience allowed our more experienced participants to better adapt to the working memory demands of the strategy game used in the study, without affecting their ability to learn the relatively attentionally demanding action game. This theory is supported by the observed contribution of *BSpan*, a measure of working memory capacity, to learning of the strategy game but not the action game. Overall, the relationship between game habits and the learning of novel games does not appear to be as straightforward as the “learning to learn” model suggests. Specifically, the learned capacity to learn new, complex tasks appears to relate differently to novel tasks that vary regarding the types of cognitive demands. In terms of video game cognition research, our results demonstrate that this claim needs to be examined with regard to the specific cognitive demands of the video game being learned.

Self-identification as a gamer (*Identification*) significantly predicted learning of the action game but not the strategy game. This relationship was stable across our wide age range and persisted even after corrected for potential gender effects. There is a well-documented positive relationship between identification as a gamer and both frequency and duration of video game play (Stone, 2019), which data from the present study reflect (Figure 2), but *Identification* importantly differs from these other measures, as it solely reflects self-perception rather than any more quantifiable habit. This effect may be explained as a matter of preference, as the action game was significantly preferred over the strategy game in our sample, and the degree of preference of the action game over the strategy game was strongly correlated with *Identification* (Figure 2). Importantly, we did not identify any gender differences in either *identification* as a gamer or genre *preference* in our sample, contrary to past research (Stone, 2019), which precludes gender as a factor explaining the relationship between gamer *identification* and *learning* of the action game.

The wide age range of our sample (18–77 years) afforded us a unique opportunity to examine the interaction between the participants’ age and their measures of learning, habit, preference, and cognition. Understanding these age-related interactions is of particular importance considering the prevalence of video game–based interventions targeting older adults (for meta-analysis, see Lampit et al., 2014; Basak et al., 2020). In the current study, age was, as expected, negatively correlated with gaming habit variables (Osmanovic and Pecchioni, 2016), as well as with measures of working memory (Bopp and Verhaeghen, 2005) and processing speed (Cerella, 1990). These age–cognition results are in line with past meta-analysis where age-related declines are significant for processing speed (Cerella, 1990) and working memory capacity (including *BSpan*), but not with short-term memory capacity indexed by *FSpan* (Bopp and Verhaeghen, 2005). Our results suggest that age-related declines are observed in processing resources and coordination between multiple items but not in maintenance of items in a temporary memory buffer. In addition to these standard neuropsychological measures of cognition, age was found to be negatively correlated with the learning of both games; this result is similar to prior studies on age-related differences in game learning (e.g., Ray et al., 2017).

It is important to note that although individual differences in age predicted cognitive and learning outcomes as well as game habits, the patterns of the predictive effects of cognition and game habits on novel game learning did not vary with age. Our results suggest that the differential, interrelated patterns of game habits, cognition, and novel game learning for action and strategy games are stable across the life span. Considering the prevalence of video game intervention as a method of cognitive intervention in older adults (Lampit et al., 2014; Toril et al., 2014; Basak et al., 2020), and the stability of these effects across the life span, these results can potentially inform expectations of intervention effects in that population. Therefore, expected gains on cognition from action versus strategy video game training, and their dose response effects (frequency, duration), may be stable across adulthood. For example, assuming these results generalize, the specific ability to learn tasks with high working memory demands may result from extended strategy game training in not only younger but also older adults. Importantly, a preference for the action versus the strategy game genre had no differential effects on learning of the two novel games, suggesting that the game-based benefits to cognition may supersede individual’s game preferences. Such generalization is far from guaranteed, however, and independent replication of these results is warranted, with particular attention paid to the cognitive profiles of the video games examined.

## Data Availability Statement

The datasets generated for this study are available on request to the corresponding author.

## Ethics Statement

This study was approved by the Institutional Review Board of the University of Texas at Dallas. All participants were provided with detailed study information, and responded with written consent, before participating in this study.

## Author Contributions

CB designed and supervised the research. ES, AH, and BB collected the data. ES and BB analyzed the data. ES and CB wrote the manuscript. All authors approved the final version of the manuscript for submission.

## Conflict of Interest

The authors declare that the research was conducted in the absence of any commercial or financial relationships that could be construed as a potential conflict of interest.
