# Corrigendum: Past Gaming Experience and Cognition as Selective Predictors of Novel Game Learning Across Different Gaming Genres

**DOI:** 10.3389/fpsyg.2021.687696

**Published:** 2021-05-10

**Authors:** Evan T. Smith, Bhargavi Bhaskar, Alex Hinerman, Chandramallika Basak

**Affiliations:** Center for Vital Longevity, The University of Texas at Dallas, Dallas, TX, United States

**Keywords:** video games, genres, learning, life span, working memory, gaming habits

In the original article, the analyses reported in Tables 3, 4, and 5 were erroneously run on an incomplete dataset (*n* = 90). The revised analyses utilizing the entire dataset for this study (*n* = 107) are presented in the revised Tables 3, 4, and 5, as presented below. The pattern of results does not differ between our originally reported statistics and those presented here.

Table 3Results of stepwise regression across 3 steps.

**Table:** 

	**Action LC Models**					**Strategy LC Models**			
**Model**	***R^**2**^***	***ΔR^**2**^***	***F***	***p***	**Model**	***R^**2**^***	***ΔR^**2**^***	***F***	***p***
1) Age + Gender	0.57	–	69.3	**<0.01**	1) Age + Gender	0.53	–	58.82	**<0.01**
2) + Identification	0.73	0.15	90.89	**<0.01**	2) + Duration	0.62	0.09	56.66	**<0.01**
3) + Duration	0.75	0.02	74.6	**<0.01**	3) + BSpan	0.64	0.02	45.45	**<0.01**

**Table:** 

	**Regression Model from Step 3**				**Regression Model from Step 3**		
**Factors**	***β***	***t***	***p***	**Factors**	***β***	***t***	***p***
Age	−0.03	−10.9	**<0.01**	Age	−0.03	−9.13	**<0.01**
Gender	−0.12	−1.25	0.2	Gender	0.02	0.15	0.89
Identification	0.19	3.47	**<0.01**	Duration	0.19	4.8	**<0.01**
Duration	0.13	2.79	**0.01**	BSpan	0.12	2.25	**0.03**

*Details of final regression model from step 3 are provided*.

*Bold values indicate p < 0.05*.

Table 4Results from follow-up regression models, after controlling for opposite game learning.

**Table:** 

	**Action LC Models**					**Strategy LC Models**			
**Model**	***R^**2**^***	***ΔR^**2**^***	***F***	***p***	**Model**	***R^**2**^***	***ΔR^**2**^***	***F***	***p***
1) Age + Gender	0.57	-	69.3	**<0.01**	1) Age + Gender	0.53	-	58.82	**<0.01**
2) + Strategy LC	0.68	0.11	73.36	**<0.01**	2) + Action LC	0.65	0.12	64.06	**<0.01**
3) + Add. Predictors	0.78	0.10	69.55	**<0.01**	3) + Add. Predictors	0.68	0.03	42.3	**<0.01**

**Table:** 

	**Regression Model from Step 3**				**Regression Model from Step 3**		
**Factors**	***β***	***t***	***p***	**Factors**	***β***	***t***	***p***
Age	−0.02	−5.95	**<0.01**	Age	−0.02	−4.23	**<0.01**
Gender	−0.12	−1.35	0.18	Gender	0.06	0.56	0.58
Strategy LC	0.28	3.65	**<0.01**	Tank LC	0.37	3.36	**<0.01**
Identification	0.18	3.45	**<0.01**	Duration	0.10	2.22	**0.03**
Duration	0.08	1.78	*0.08*	BSpan	0.09	1.77	*0.08*

*Additional predictors in Step 3 were significant predictors from the previous step-wise regressions described in Table 3*.

*Bold values indicate p <0.05; Italicized values indicate p < 0.10*.

Table 5Results of moderator analyses.

**Table:** 

	**Action LC Models**					**Strategy LC Models**			
**Model**	***R^**2**^***	***ΔR^**2**^***	***F***	***p***	**Model**	***R^**2**^***	***ΔR^**2**^***	***F***	***p***
1) Gender	0.01	–	1.2	0.28	1) Gender	0.01	–	0.09	0.77
2) + Age & Add. Preds.	0.75	0.74	74.59	**<0.01**	2) + Age & Add. Preds.	0.64	0.63	45.45	**<0.01**
3) + Moderators	0.76	0.01	51.34	**<0.01**	3) + Moderators	0.65	0.01	30.36	**<0.01**

**Table:** 

	**Regression Model from Step 3**				**Regression Model from Step 3**		
**Factors**	***β***	***t***	***p***	**Factors**	***β***	***t***	***p***
Gender	−0.11	−1.12	0.27	Gender	0.02	0.18	0.85
Age	−0.03	−9.17	**<0.01**	Age	−0.02	−6.34	**<0.01**
Identification	0.19	3.48	**<0.01**	Duration	0.21	4.82	**<0.01**
Duration	0.15	2.98	**<0.01**	BSpan	0.13	2.48	**0.02**
Age * Identification	−0.14	−1.89	*0.09*	Age * Duration	0.09	1.07	0.29
Age * Duration	0.16	1.74	*0.06*	Age * BSpan	0.04	0.69	0.49

*Bold values indicate p < 0.05; Italicized values indicate p < 0.10*.

The authors apologize for this error and state that this does not change the scientific conclusions of the article in any way. The original article has been updated.

